# Risk of Progressive Multifocal Leukoencephalopathy in Multiple Sclerosis Patient Treated With Natalizumab: A Systematic Review

**DOI:** 10.7759/cureus.14764

**Published:** 2021-04-30

**Authors:** Govinathan Vivekanandan, Ansha P Abubacker, Revathi Myneni, Harsh V Chawla, Aimen Iqbal, Amit Grewal, Andrew Ndakotsu, Safeera Khan

**Affiliations:** 1 Internal Medicine, California Institute of Behavioral Neurosciences and Psychology, Fairfield, USA; 2 Emergency Medicine, California Institute of Behavioral Neurosciences and Psychology, Fairfield, USA

**Keywords:** progressive multifocal leukoencephalopathy, natalizumab, monoclonal antibody

## Abstract

Natalizumab, a monoclonal antibody acting on alpha4 integrin receptors, is frequently used to treat multiple sclerosis patients. The biggest downside is the risk of development of progressive multifocal leukoencephalopathy, an immune-related condition affecting mainly the central nervous system. The presence of the John Cunningham virus (JCV) and its reactivation is an important factor in the development of progressive multifocal leukoencephalopathy (PML). This study highlights its different proposed mechanism and risk factors strongly related to natalizumab-induced progressive multifocal leukoencephalopathy. The pieces of literature will also be reviewed to look for a relation between the JCV and natalizumab-induced progressive multifocal leukoencephalopathy in multiple sclerosis treated patients.

The articles were searched from three databases and reviewed systematically. The inclusion criteria for this study were patients aged 20-50 years, English language paper, full-text availability, and human studies, whereas articles on patients with AIDS and cancer-related disease prior to natalizumab treatment were excluded.

Out of 6531 articles identified after applying the search strategy on three main databases PubMed, Google Scholar, and ResearchGate, a total of 32 articles were finalized for the review. This study follows the guidelines listed in the Preferred Reporting Items for Systematic Reviews and Meta-Analyses (PRISMA) checklist 2009. The data collected from these finalized articles were pertaining to the risk factor related to natalizumab induced progressive multifocal leukoencephalopathy and the mechanism related to its pathogenesis.

Natalizumab is known to have the potential to cause progressive multifocal leukoencephalopathy in treated patients; here, we evaluate a close relationship of its related risk factors. The articles studied exhibit a close relationship between the length of natalizumab treatment and the presence of the JCV before infusion of natalizumab. From our analysis, it seems that the mechanism related to natalizumab-induced PML is strongly related to antigen-specific T cells and its effects. The frequency of monitoring and vigilance on the management of patients treated with natalizumab will help detect progressive multifocal leukoencephalopathy.

## Introduction and background

The global incidence of progressive multifocal leukoencephalopathy (PML) in natalizumab treated patients is 4.08/1000 patients (95% confidence interval (CI) 3.80-4.36/1000 patients, and the survival rate is approximately 70-75% [1,2]. In November 2004, the approval of natalizumab in managing multiple sclerosis (MS) by the FDA was accelerated based on one-year results from two randomized, placebo-controlled phase three clinical trials, the affirm and sentinel trials [2]. However, the drug was removed from the market in February 2005, and the active ongoing trials were terminated due to the two reported cases of PML in the sentinel trial [3]. Natalizumab was reintroduced into the market in June 2006 with FDA approval as a monotherapy in relapsing-remitting multiple sclerosis. The drug required new labeling and the safety warnings were upgraded to clarify the potential risk of PML [3].

PML is a diffuse demyelinating disease of the white matter of the brain due to the John Cunningham virus (JCV) and its infection on oligodendrocytes creating a widespread lesion in the brain [3]. JCV, a human polyomavirus 2, got its name as it was first isolated from John Cunningham in 1971 [3]. The approach to the diagnosis of PML has evolved since its initial description in 1958 [4]. PML diagnosis is by the neuropathological demonstration of the histopathological triad (demyelination, bizarre astrocytes, and enlarged oligodendroglia nuclei) [4]. Clinical MRI coupled with the JCV demonstration by cerebrospinal fluid polymerase chain reaction (PCR) is also a diagnostic approach [4]. The bulk of PML-related cases occur in patients with compromised cell-mediated immunity, and to date, there is no distinct management for PML.

In 2005, PML was reported in multiple sclerosis (MS) patients treated with natalizumab [3]. Multiple sclerosis a chronic demyelinating disease of white matter in the central nervous system where autoreactive lymphocyte activity has been strongly associated with its pathophysiology [5]. In the past 10 years, a strong input on the mechanism of relapsing-remitting MS has led to the recent development of different-disease modifying therapies, lessening both ferocity and periodicity of new relapses by altering or suppressing the immune system [5]. The proposed mechanism of action of Natalizumab on PML was focused on cellular mediated immunity and their actions [6].

In view of natalizumab (Tysabri)-induced PML, Tysabri is only available via a risk evaluation and mitigation strategy (REMS) program in the United States [7]. Studies on biomarkers identifying a patient at risk of developing PML in natalizumab treated patients and recent advancements on the use of better MRI to detect PML in early stages are being studied [8,9].

In the current century, there are still gaps related to natalizumab-associated progressive multifocal leukoencephalopathy. Early asymptomatic or non-specific manifestations coupled with the lack of cure have posed a significant challenge for physicians worldwide in treating MS patients with natalizumab. The dilemma of balancing monoclonal antibody's effectiveness in relapsing-remitting multiple sclerosis (RRMS) and the fatal non-curable side-effect of the drugs in the market has been growing among internists.

Recent safety campaigns on safety and guidelines on how to use natalizumab in MS patients have not changed the fact that the incidence of natalizumab associated with PML and patient's lack of confidence in the drug is growing. The lack of insight on PML and its association with monoclonal antibodies, especially natalizumab, which leads to poor compliance, is the main concern to be addressed here. This study will detail the risk of progressive multifocal leukoencephalopathy in multiple sclerosis patients treated with natalizumab and other monoclonal antibodies and its pathogenesis.

## Review

Progressive multifocal leukoencephalopathy is a fatal yet less understood disease in the current generation. The article is focused on the risk of natalizumab and its pathophysiology on PML. All articles collected were reviewed systematically and their methods and result are explained in detail.

Method

We searched for scientific research articles to answer the question, "Do natalizumab and other monoclonal antibodies cause PML in multiple sclerosis-treated patients?" As a guide, Preferred Reporting Items for Systematic Review and Meta-Analyses (PRISMA) 2009 Guidelines were used throughout the study process. Articles were searched from three databases PubMed, Google Scholar, and ResearchGate using specific keywords pertaining to the research topic. The keywords are as follows: progressive multifocal leukoencephalopathy, natalizumab, and monoclonal antibodies. The medical subject headings (MeSH) is the National Library of Medicine (NLM) control vocabulary thesaurus which was specifically used to search articles in PubMed databases. The final search strategy used was as follows: "("Leukoencephalopathy, Progressive Multifocal/blood"[Mesh] OR "Leukoencephalopathy, Progressive Multifocal/cerebrospinal fluid"[Mesh] OR "Leukoencephalopathy, Progressive Multifocal/diagnosis"[Mesh] OR "Leukoencephalopathy, Progressive Multifocal/diagnostic imaging"[Mesh] OR "Leukoencephalopathy, Progressive Multifocal/drug therapy"[Mesh] OR "Leukoencephalopathy, Progressive Multifocal/epidemiology"[Mesh] OR "Leukoencephalopathy, Progressive Multifocal/etiology"[Mesh] OR "Leukoencephalopathy, Progressive Multifocal/history"[Mesh] OR "Leukoencephalopathy, Progressive Multifocal/immunology"[Mesh] OR "Leukoencephalopathy, Progressive Multifocal/pathology"[Mesh] OR "Leukoencephalopathy, Progressive Multifocal/physiology"[Mesh] OR "Leukoencephalopathy, Progressive Multifocal/physiopathology"[Mesh]) AND ("Natalizumab/administration and dosage"[Mesh] OR "Natalizumab/adverse effects"[Mesh] OR "Natalizumab/blood"[Mesh] OR "Natalizumab/cerebrospinal fluid"[Mesh] OR "Natalizumab/drug effects"[Mesh] OR "Natalizumab/etiology"[Mesh] OR "Natalizumab/immunology"[Mesh] OR "Natalizumab/organization and administration"[Mesh] OR "Natalizumab/pharmacology"[Mesh] OR "Natalizumab/therapeutic use"[Mesh] OR "Natalizumab/toxicity"[Mesh]) AND ("Antibodies, Monoclonal/adverse effects"[Mesh] OR "Antibodies, Monoclonal/cerebrospinal fluid"[Mesh] OR "Antibodies, Monoclonal/drug effects"[Mesh] OR "Antibodies, Monoclonal/immunology"[Mesh] OR "Antibodies, Monoclonal/therapeutic use"[Mesh] OR "Antibodies, Monoclonal/toxicity"[Mesh]).” Other databases used with their keywords are mention in Table 1.

**Table 1:** Databases and search result

Databases	Keywords	Search result
PubMed	Final strategy as stated above	169
Google Scholar	Progressive multifocal leukoencephalopathy and natalizumab and monoclonal antibody	6260
ResearchGate	Progressive multifocal leukoencephalopathy and natalizumab and monoclonal antibody	102

Eligibility Criteria

The screening was done for the title and abstract-related searches in which only multiple sclerosis-treated patient was chosen. We only chose the papers published in the English language, and both clinical-trials and non-clinical-trials-related papers were selected. Articles related to animal study, in general, were excluded from this study. Patients in middle ages (20-50 years) were chosen. All patients affected by AIDS due to HIV or cancer-related diseases before treatment with natalizumab were eliminated from the studies. These exclusion criteria were set in order to get specific articles related to natalizumab and its risk factors in patients who were not immunosuppressed prior to treatment as this can create bias in our discussion. 

Result

**Figure 1:**
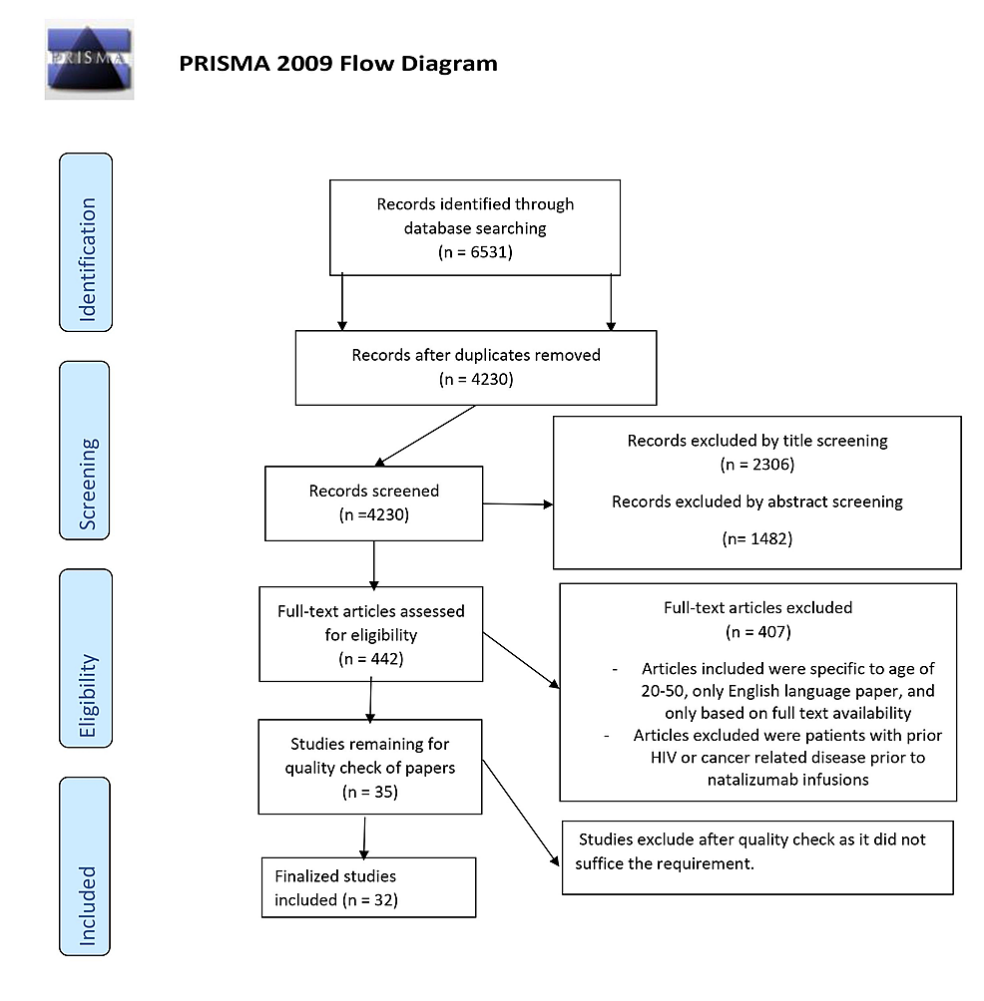
PRISMA flow diagram PRISMA: Preferred Reporting Items for Systematic Review and Meta-Analyses

A total of 6531 articles were collected using the search strategy mentioned in the method section above and were then screened based on the title and abstract related to the risk of PML in natalizumab and monoclonal antibody-treated multiple sclerosis patients. Papers were also filtered based on the eligibility criteria and availability of full text. Results show only 35 items were available, and this article was a check for quality based on its study characteristics. A complete PRISMA flow diagram is created (Figure 1).

For randomized control trials papers, the Cochrane risk-of-bias tool was used. For case reports, a systematic review and observational study, the Joanna Bricks Institute (JBI) Critical Appraisal tool, the Prisma checklist, and the Newcastle Ottawa Scale were used. Based on the finalized study collected, six were case reports, seven were observational studies, three were randomized control trials, and 18 were systematic reviews with two meta-analyses. A total of 1153 patients were observed in this study, where most were studied before natalizumab infusion. Others were studied when patients are already on natalizumab to assess for risk stratification of PML. Gender specificity was not mentioned in the articles reviewed; thus, they could not be used as a comparison in this study.

Discussion

Recombinant humanized monoclonal immunoglobulin antibodies affecting alpha-4 integrin in relapsing-remitting multiple sclerosis were first studied with the short-term placebo-controlled trial. Subsequently, bigger tests were conducted. Sentinel trial conducted in 2007 shows two cases of PML, of which one was fatal [10].

Natalizumab-Induced PML and Its Pathogenesis

A post-mortem study by Wüthrich et al. of an MS patient showed extensive PML lesion in the subcortical and deep white matter of both hemispheres, corpus callosum, and the brainstem [11]. Macroscopically lesion was identified as softened, granular and moth-eaten appearance compared to MS lesion [11]. Specimen were also microscopically analyzed, showing significant myelin loss with a high density of infected oligodendrocytes and astrocytes [11]. On further review, it shows the highest risk for development of PML comes from monoclonal antibody natalizumab resulting in the release of CD34+ cells from bone marrow [11,12]. Based on few case studies reviewed it is not clear whether CD34+ progenitor cells mediate JC viremia inducing PML. 

Few case reports have been published on PML-induced natalizumab. These case reports are summarized. These cases show evidence that natalizumab therapy induces PML lesions in multiple sclerosis patients and the need for early screening and a more cautious approach in management and guidelines.

First case summary: A 52-year-old woman diagnosed as having relapsing-remitting multiple sclerosis of 14 years duration has been treated with several immunomodulatory drugs. In November 2006, 300mg of natalizumab was given intravenously every four weeks. It was given for refractory exacerbations. In June 2009, she presented with cognitive decline, magnetic resonance imaging was conducted and a new left frontal subcortical non-enhancing white matter lesion in addition to the stable MS lesion was seen. Her symptoms worsen and subsequent MRI findings show the marked progression of the lesion. PCR JCV DNA from cerebrospinal fluid was detected positive. Natalizumab was stopped in September 2009, she was given a course of plasma exchange but due to severe progression of PML, she demised of aspiration pneumonitis in November 2009, four months after PML onset, two months after the last natalizumab injection [11].

Second case summary: A 44-year-old female patient with typical relapsing-remitting multiple sclerosis has been on natalizumab for 62 months after severe disease progression despite interferon-beta1a. PML suspicious findings were detected incidentally during routine magnetic resonance imaging in a clinically stable patient. A specific lesion spread through the brain. The patient did not consent to JCV DNA testing in the early stage. The MRI findings were then confirmed as PML lesions on repeated MRI examination [12]. Dosage of natalizumab and its pathology were not mentioned in the case study review.

Third case summary: A 26-year-old woman with no significant medical history was diagnosed with multiple sclerosis in 2013 at the age of 22 years. She was started with glatiramer acetate and dimethyl fumarate then transitioned to natalizumab in July 2014 and her JCV antibody index was 3-58 prior to natalizumab therapy. An asymmetric confluent non-enhancing hyperintensity in the bilateral subcortical pre-central gyri which is highly consistent with progressive multifocal leukoencephalopathy was seen in a surveillance MRI in November 2016. PCR JCV DNA was positive and a diagnosis was made. Natalizumab was discontinued after 27 total treatments. Three months after diagnosis she noticed mild dysmetria that progressed to tremor, repeated brain MRI shows enhancing lesion suggesting immune reconstitution inflammatory syndrome (IRIS). She was then given methylprednisolone for IRIS and started back on glatiramer acetate [13].

**Figure 2:**
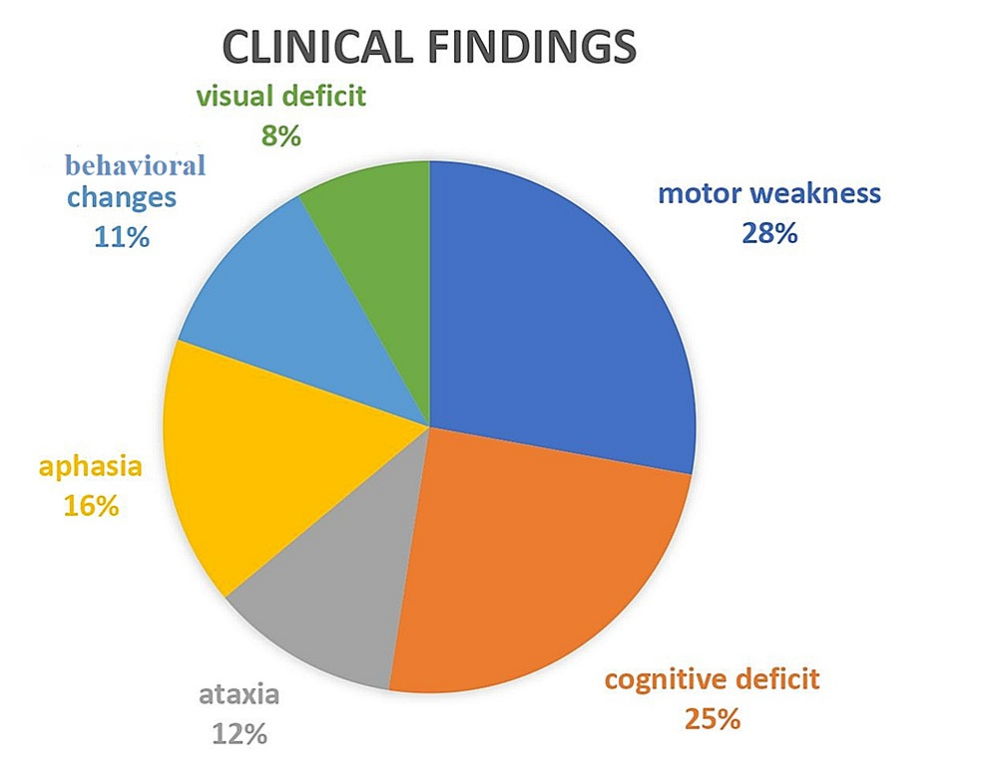
Clinical findings Motor weakness: monoparesis, hemiparesis, tetraplegia, monoplegia Cognitive deficit: confusion Aphasia: true aphasia and word-finding difficulty Behavioral changes: personality changes, apathy, lethargy, agitation Visual deficit: visual field defect

Based on the studies reviewed on 87 MS patients treated with natalizumab, the most common symptoms/sign are motor weakness, cognitive deficit, ataxia, aphasia, behavioral changes, and visual deficit [14]. Clinical findings are depicted in Figure 2.

**Figure 3:**
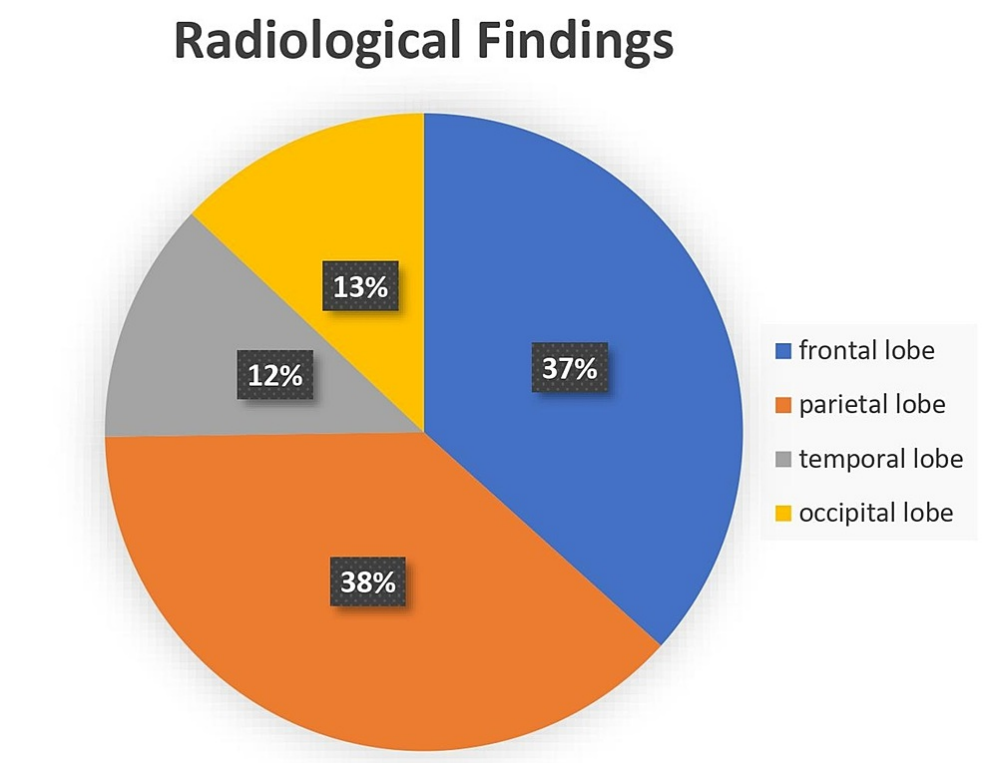
Radiological findings

Natalizumab-associated PML is more prevalent in the supratentorial area, evident radiologically with MRI [14]. Lesions most commonly involve the frontal and parietal lobes, whereas temporal and occipital are least involved [14]. These findings are depicted in Figure 3.

Based on these case reports, there are no defining early physical signs to detect PML and only via MRI. The data show the duration of treatment inducing PML varies with patients, but all is certain is that we still do not have a cure for PML lesions caused by natalizumab. Do early screening helps detect PML lesion, and when should we start screening, and if detected what we can do to stop the lesion from progressing as there is no cure till date. The limitations seen from this review are that we only reviewed one post-mortem study regarding the histopathological findings. There could be other lesions presenting at different sites of the central nervous system that we are unaware of. The review from this post mortem study is related to one patient; thus, the study's power is low. Early MRI findings are an important tool in detecting PML, and its benefit is not reviewed in these articles.

JCV and Immunotoxicity in Patients Treated With Natalizumab

JCV or human polyomavirus 2, named after its detection in a patient named John Cunningham (JC). It was first identified by electron microscopy in 1965 by Zuhrein and Chou and Silverman and Rubinstein [15,16]. Two sites of entry for the JCV have been suggested, which are tonsil and gastrointestinal. Primary viremia is symptomless and occurs in childhood. Initial infection remodels into neurotropic form by gene rearrangement and successfully reproduces in glial tissues. The mode of transmission of the JCV is still not defined to date. 

An important role in virus replication as a nuclear localization signal in T antigen to the nucleus and the helicase in it unwinds the helix to start viral DNA replication [17]. As discussed, natalizumab prevents inflammatory cells from crossing into the blood-brain barrier and other tissue. Vascular leukocyte adhesion four (VLA4) (a4 integrin) are used to bind with vascular cell adhesion molecule (VCAM). It shows in MS patients treated with natalizumab CD4+, CD8+, CD 19+ B cells, and CD 138+ plasma at lower CSF levels than controls [6]. There are three proposed mechanisms by authors from the studies reviewed [6,17,18]. These theories show how natalizumab is tied to PML and its effect on the body's immune defense. The first proposed mechanism of action shows that natalizumab has a sequel on T cells which respond against the vigorous and dormant JCV [6]. JCV-specific T cells are important in containing PML and these cells are significant mostly in the central nervous system(CNS) but also in the bone marrow and spleen [6]. Natalizumab prevents the entry of these cytotoxic T cells into brain tissue/other sites where JCV may be dormant thus averts JCV subduing [6].

The second proposed mechanism of action shows the focus and retention of lymphocytes in the bone marrow and spleen are mediated by a4B1 VCAM [6]. Natalizumab blocks a4B1 integrin receptors thus leads to the release of B cells from bone marrow and spleen, which is the common site of JCV latency [6]. This leads to an increase in antigen-specific T cells and reactivation of latent JCV [6,18]. This proposed theory is widely supported by the articles reviewed [6,17,18]. The third proposed theory relies on a previously reported case of PML after natalizumab therapy involving a patient who had undergone simultaneous treatment with either interferon B or other immunosuppressant. This shows that a quell immune condition is needed for the evolution of PML in patients treated with natalizumab [6]. This statement is not conclusive, as stated by the case review one patient with natalizumab without prior immunosuppressant can develop PML [11].

The anti-JCV antibody has been recognized to diagnose reactivation of JCV in PML, but its activation is not seen in an early stage of disease [18]. Early biomarkers to detect PML in its early stage after administration of natalizumab have yet been discovered. These early biomarkers will help develop new guidelines in managing MS patients with natalizumab and better guidance to the physician to prevent PML lesions' development. There is a limitation to these articles reviewed as there was a minimal study conducted in JCV-negative patients to compare the significance of positive JCV in natalizumab-induced PML and data collected is small in number.

Natalizumab on Withdrawal and PML Risk Stratification

Immune reconstitution inflammatory syndrome (IRIS) occurs not only in patients with post highly active retroviral treatment (HAART) treatment but also with natalizumab associated progressive multifocal leukoencephalopathy after withdrawal. Upon withdrawal, a paradoxical inflammatory reaction is probably an exaggerated response to the JCV by activating the host immune system [19]. In a study, IRIS was developed in almost all patients treated with natalizumab which has caused PML consequent cessation of natalizumab and removal from the bloodstream by plasma exchange [20]. Twenty-eight cases between 2006 and 2009 have described PML and IRIS features [21].

The rapid deterioration of neurological deficit with MRI evidence of lesion progression with contrast enhancement has been the clinical picture reported. In a study of 39 patients, 27 had PML IRIS after 82.5 days (range of 53.3 to 111.7 days) following natalizumab withdrawal. The same study also shows prior immunosuppressive agents before natalizumab treatment have no relation with the current development of PML-IRIS after natalizumab withdrawal [22]. Withdrawal of PML can be due to many reasons, whether it is due to physician's lack of continuous monitoring or patients' lack of compliance; either way, the development of IRIS can sometimes be fatal.

Vermerson et al. demonstrated that the diagnosis of PML varies between fatal and non-fatal is 62.8 days and 44.2 days, respectively [23]. In a study cohort, it shows non-fatal PML diagnosis interval is 30 days [23]. JCV antibody status, immunosuppressive pre-treatment, and natalizumab therapy duration are the current accepted criteria as factors in risk stratification to natalizumab induced PML.

Immunosuppressives pre-treatment: The risk of developing PML is three to four times higher in patients with prior use of immunosuppressants. A study shows various immunosuppressant use with their significant risk in developing PML after natalizumab induction [23]. The result is tabulated in Table 2.

**Table 2:** Immunosuppressants used before natalizumab treatment with its relation to progressive multifocal leukoencephalopathy and its incidence

Immunosuppressants	Incidence of progressive multifocal leukoencephalopathy cases upon natalizumab treatment
Mitoxantrone	36/68 (55.9%)
Cyclophosphamide	14/68 (20.6%)
Azathioprine	11/68 (16.2%)
Methotrexate	9/68 (13.2%)
Mycophenolate mofetil	6/68 (8.8%)
Others	8/68 (11.8%)

The risk of PML is 0.31/1000 in immunosuppressant naïve versus 0.88/100 with prior immunosuppressants makes it clear that immunosuppressant does play its part in the development of PML [24]. The question to arise is how the mechanism of action of these various drugs interlinked with natalizumab or other monoclonal antibodies in the development of PML lesions and whether the latency of PML is shortened due to these immunosuppressants. As this is not within the scope of this review, we cannot make any comments regarding this.

Duration of natalizumab therapy: Strong relation has been developed between the duration of natalizumab therapy and the development of PML lesions. A review from a study conducted has shown low incidence during the first treatment year with 0.06 (95% confidence interval (CI) 0.02-0.12), which increases during the second year with 0.67 (95% CI 0.51-0.86). In the third year of treatment, the incidence has dramatically spiked up with 1.84 (95% CI 1.53-2.21), and a year after that, which is 48 months of natalizumab therapy, has shown the highest incidence 2.36 (95% CI 1.82-2.92). The fifth year of the study shows an incidence of 2.33 (95% CI 1.82-2.92) [23]. This data collected shows the importance of detecting PML in its early asymptomatic stage as lack of detection in early days significantly increased the risk of developing PML.

Anti-JCV antibody serostatus: As discussed, the JCV has a positive correlation with progressive multifocal leukoencephalopathy. Anti-JCV antibodies are used worldwide as an indicator to aid in the risk stratification of natalizumab-induced PML. In a study, patients were stratified into two index groups less than 1.5 and more than 1.5 anti-JCV antibody index values [24]. It is shown that an index value of <1.5 has a lower PML risk depending on the duration of natalizumab therapy [24]. An index value less than 1.5 carry a 0.71/1000 (within 1-24 months natalizumab therapy) incidence of PML, less than 0.9 carries a risk of 0.51/1000 (within 25-48 months of treatment), which rises to 1.13/1000 if the index value is an increase to 1.5. PML risk with index >1.5 increase dramatically to 8.83/1000 (within 25-48 months) and increase to 10.12/1000 (within 49-71 hours of natalizumab treatment) [24].

This shows that these three factors do not just work independently but are closely related to one another. Some countries have used these criteria to treat the patient with MS treated with natalizumab to reduce the incidence of PML. As these guidelines were not studied by the articles reviewed here, we cannot comment on this, but more data is being collected on this guideline and its effectiveness [24]. Studies have also shown that both physician and patient's knowledge on this disease is low [25].

As guidelines are different worldwide in managing this condition, such consistent guidelines must exist, and most importantly, adhere to them. As reviewed, the consequences of ignoring the early symptomatic signs, or misinterpreting them, or even the failure to monitor patients on natalizumab treatment could lead to severe consequences and even death. A new body supervising physicians worldwide should be developed to monitor the incidence of PML, the frequency of follow-up, and consensus guidelines should exist in detecting and managing PML. This will strategize the need for more research study into the early detection of the PML in natalizumab and monoclonal antibody-treated MS patient and reducing its morbidity as the effectiveness of natalizumab in MS patients is proven to date.

Based on the studies reviewed, we will like to propose to include early MRI screening in all patients treated with natalizumab unless its contraindicated, and the possibilities of 'drug holiday' in the duration of treatment with natalizumab should be studied in the future. The limitations in our study, are on the immunosuppressive agent used before natalizumab treatment. As our exclusion criteria excluded this factor, the studies collected might show bias to this factor. Different types of immunosuppression might or might not play their role in inducing PML based on their mechanism of action.

## Conclusions

Progressive multifocal leukoencephalopathy, a fatal disease, is a risk in multiple sclerosis patients being treated with natalizumab or other monoclonal antibodies. Its pathogenesis involves the drug natalizumab and its action on alpha4 integrin allowing reactivation of the John Cunningham virus leading to PML lesion in the brain. The frontal and parietal lobes have been the most common site of lesion to look for in the early stage of the disease. The relation of the JCV and PML in natalizumab treated patients has been clearly defined. Still, the possibility of progressive multifocal leukoencephalopathy developing in non-JCV patients has to be studied in more detail. The focus on PML risk stratification must be taken with high scrutiny while the patient is on natalizumab or other monoclonal antibodies. The need for frequent monitoring is understated. The factors like duration of treatment and JCV positivity show close relation and more studies related to drug latency can be useful in reducing PML lesions.

This paper exhibits the significant risk of PML and shows that more attention is needed to manage multiple sclerosis patients treated with natalizumab. Physicians need to be more cautious and vigilant in detecting early signs of progressive multifocal leukoencephalopathy as much as patient compliance matters. Proper worldwide standardized guidelines are necessary for physicians, to reduce the morbidity and mortality of natalizumab-induced PML. As monoclonal antibodies are becoming the drug of choice for many immune-related diseases, it is important to research more of its side effect and prevent its complications.
